# A New Method in Incarcerated Scrotal Hernia

**Published:** 1886-11

**Authors:** W. A. Lockett

**Affiliations:** Brenham, Texas


### A NEW METHOD IN INCARCERATED SCROTAL HERNIA.

*Editor Daniel's Texas Medical Journal:*

THE patient, a large stout man about 35 years old, had been suffering from complete strangulation for twenty-four hours. The scrotal sack was as large as an infant's head at a year old, very hard and tense. Taxis, the knee-chest position, suspension with head down, and various other methods had been tried in vain. His condition was becoming serious. I had in my pocket a Martin's India rubber bandage. I took the scrotum in my left hand to steady it, and began with the band at the distal end to strap, making strong tension, and as the strapping approached the canal in a spiral manner, decreased the tension. I then wound the band back to the distal end, increasing the tension. In five minutes of this steady and expulsive pressure, I had the satisfaction of seeing the sack diminishing, and suddenly the whole collapsed with a gurgle, and the reduction was complete.

Now, Mr. Editor, if this is worth putting in your journal, you may do so; if not, put it in the waste basket.

W. A. Lockett, M. D.,
Brenham, Texas.

